# MicroRNA2871b of Dongxiang Wild Rice (*Oryza rufipogon* Griff.) Negatively Regulates Cold and Salt Stress Tolerance in Transgenic Rice Plants

**DOI:** 10.3390/ijms241914502

**Published:** 2023-09-25

**Authors:** Wanling Yang, Yong Chen, Rifang Gao, Yaling Chen, Yi Zhou, Jiankun Xie, Fantao Zhang

**Affiliations:** 1 College of Life Sciences, Jiangxi Normal University, Nanchang 330022, China; yangwl3058@163.com (W.Y.); chenyong05@gmail.com (Y.C.); 18078500774@163.com (R.G.); yaqing620@163.com (Y.C.); zhouyi25@mail2.sysu.edu.cn (Y.Z.); xiejiankun11@163.com (J.X.); 2 State Key Laboratory of Crop Gene Exploration and Utilization in Southwest China, Sichuan Agricultural University, Chengdu 611130, China

**Keywords:** common wild rice, miR2871b, cold stress, salt stress, genetic resource

## Abstract

Cold and salt stresses are major environmental factors that constrain rice production. Understanding their mechanisms is important to enhance cold and salt stress tolerance in rice. MicroRNAs (miRNAs) are a class of non-coding RNAs with only 21–24 nucleotides that are gene regulators in plants and animals. Previously, miR2871b expression was suppressed by cold stress in Dongxiang wild rice (DXWR, *Oryza rufipogon* Griff.). However, its biological functions in abiotic stress responses remain elusive. In the present study, miR2871b of DWXR was overexpressed to investigate its function under stress conditions. When miR2871b of DWXR was introduced into rice plants, the transgenic lines were more sensitive to cold and salt stresses, and their tolerance to cold and salt stress decreased. The increased expression of miR2871b in rice plants also increased the levels of reactive oxygen species (ROS) and malondialdehyde (MDA); however, it markedly decreased the activities of peroxidase (POD), superoxide dismutase (SOD), and catalase (CAT) and the contents of proline (Pro) and soluble sugar (SS). These data suggested that miR2871b of DXWR has negative regulatory effects on cold and salt stress tolerance. Meanwhile, 412 differentially expressed genes (DEGs) were found in rice transgenic plants using transcriptome sequencing, among which 266 genes were up-regulated and 146 genes were down-regulated. Furthermore, the upstream *cis*-acting elements and downstream targets of miR2871b were predicted and analyzed, and several critical acting elements (ABRE and TC-rich repeats) and potential target genes (*LOC_Os03g41200*, *LOC_Os07g47620*, and *LOC_Os04g30260*) were obtained. Collectively, these results generated herein further elucidate the vital roles of miR2871b in regulating cold and salt responses of DXWR.

## 1. Introduction

Rice (*Oryza sativa* L.) is an important food crop, and more than half of the world’s population consumes it as a staple food [1]. However, the growth and development of rice plants are often threatened by multiple abiotic stresses, such as heavy metals, salt, drought, heat, and cold, which can severely affect yield and productivity [2,3]. Among these stresses, cold and salt are the major factors that significantly affect the entire life cycle of rice plants [4,5,6]. To adapt to stress conditions, a variety of adaptive regulators were induced. These regulators either function directly or participate in the regulation of the genes involved in rice stress signal transduction [7]. Therefore, elucidating the mechanism of the regulators related to cold and salt stress responses would help breed stress-adapted rice varieties.

MicroRNAs (miRNAs) are among the most important regulators of gene expression and play important roles in a variety of biological processes, including the regulation of plant growth, development, and responses to biotic and abiotic stresses [8,9,10,11]. The mechanism of miRNA regulation of gene expression is to repress gene expression by complementing it with target transcript sequences, which leads to degradation or translational repression of target genes [12,13]. Recently, the crucial roles of various miRNAs in the regulation of cold or salt stress tolerance have been validated and elucidated via overexpression experiments in various plant species [14,15,16,17,18]. For example, overexpression of tomato (*Solanum lycopersicum* L.) miR396a-5p can enhance salinity, drought, and cold stress tolerance in tobacco (*Nicotiana tabacum* L.) [19]. In *Arabidopsis thaliana*, overexpression of miR397 and miR399f increases cold and salt stress tolerance, respectively [20,21]. In rice, multiple miRNAs, such as miR319 [22], miR528 [23], miR156k [24], miR393 [25], miR1861h [26], miR396c [27], and miR171c [28], can regulate cold or salt stress response. Among these, overexpression of miR319, miR528, miR1861h, and miR393 enhances cold or salt tolerance, whereas overexpression of miR156k, miR171c, and miR396c decreases tolerance. Thus, miRNAs have been considered valuable candidates for genetically modifying rice tolerance to cold or salt stress.

Dongxiang wild rice (DXWR, *Oryza rufipogon* Griff.) is the most northerly (28°14′ N) wild rice germplasm resource found worldwide [29,30,31,32]. DXWR has numerous benefits such as high yield and biotic and abiotic stress resistance [33,34]. In particular, its cold stress resistance is rare, and it can survive at low temperatures of −12.8 °C [35,36]. Therefore, mining stress-responsive miRNAs from DXWR and further exploring their molecular mechanisms will be beneficial to the subsequent improvement of rice cultivars. Previously, Jiang et al. [37] revealed that the miR2871b expression was significantly suppressed by cold stress in DXWR, using high-throughput sequencing technology. This indicates that miR2871b may be associated with abiotic stress response. However, further research on miR2871b regarding the regulation of abiotic stress responses in DXWR is still required.

Therefore, we employed a transgenic approach through overexpression of DXWR miR2871b in transgenic rice plants. We analyzed the effect of miR2871b on rice plant response to cold and salt stresses by evaluating the phenotype and related physiological parameters. Meanwhile, transcriptome sequencing was conducted to investigate the underlying molecular mechanisms of miR2871b. Furthermore, the upstream regulatory elements and downstream target genes of miR2871b were predicted and analyzed. Our goal was to verify whether miR2871b of DXWR has an impact on the stress tolerance in rice plants.

## 2. Results

### 2.1. Cold and Salt Stress Decreases miR2871b Expression

**Figure 1:**
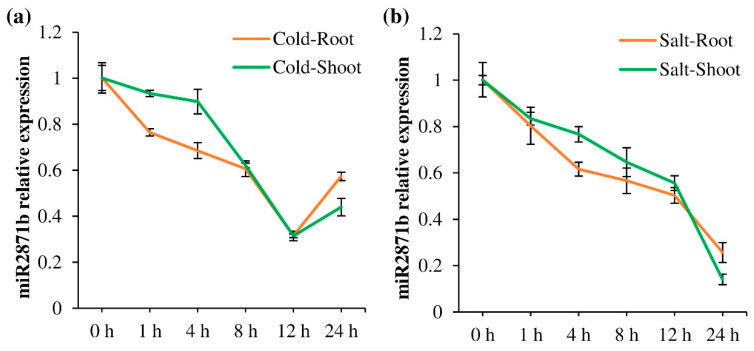
Expression pattern of miR2871b under cold and salt stress. (**a**) Expression pattern of miR2871b under cold stress. (**b**) Expression pattern of miR2871b under salt stress. Error bars show the standard deviations.

To study the expression pattern of miR2871b in response to cold and salt stress, the expression of miR2871b was analyzed by qRT-PCR. The seedlings of DXWR were treated with a low temperature of 4 °C and 200 mM NaCl. RNA samples of seedlings were extracted at six time points (0, 1, 4, 8, 12 and 24 h) of treatments. The results showed that the expression of miR2871b in the root and shoot exhibited a clear down-regulation trend under cold and salt stress treatment (Figure 1). Under cold treatment, the expression of miR2871b was down-regulated and reached the lowest level at 12 h in both root and shoot, after which expression increased somewhat (Figure 1a). Under salt treatment, the expression of miR2871b gradually decreased with the increase of treatment time in both root and shoot (Figure 1b). These results suggested that miR2871b was responsive to cold and salt stress.

### 2.2. Expression of miR2871b Was Up-Regulated in Transgenic Plants

**Figure 2:**
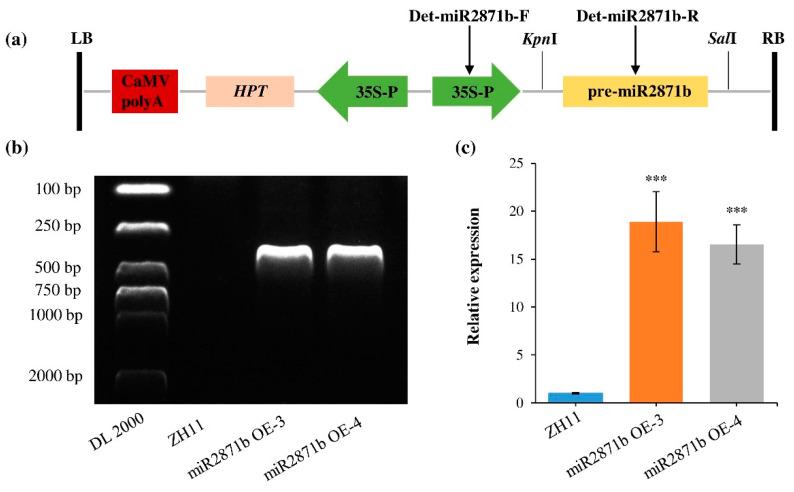
Characterization of the miR2871b transgenic rice plants. (**a**) Schematic representation of the miR2871b overexpressing construct used for *A. tumefaciens*-mediated transformation. (**b**) Genomic PCR analysis showing the presence of miR2871b overexpression construct in transgenic lines (miR2871b OE-3, miR2871b OE-4). Wild-type plants (ZH11) were used as a negative control. (**c**) Expression of miR2871b in wild-type plants and transgenic lines. Error bars show the standard deviations of the mean (*** *p* ≤ 0.001 using Student’s *t*-test).

At present, genetic transformation in wild rice is difficult and the efficiency is very low. Therefore, to investigate the roles of miR2871b in the stress response, an overexpression vector expressing miR2871b was constructed and introduced into the rice variety ZH11 via *Agrobacterium*-mediated plant transformation (Figure 2a). Transgenic rice lines of miR2871b were confirmed by hygromycin selection and PCR amplification with specific primers (Det-miR2871b-F, Det-miR2871b-R) (Figure 2b). The positions of the specific primers are indicated in Figure 2a. Moreover, the qRT-PCR results showed that the expression levels of miR2871b were significantly increased in the homozygous positive transgenic lines (miR2871b OE-3 and miR2871b OE-4) (Figure 2c), confirming the success of transgenic approaches. Subsequently, the two positive transgenic lines were used for phenotypic, physiological, and biochemical analyses.

### 2.3. Overexpression of miR2871b in Rice Reduces Cold Tolerance

**Figure 3:**
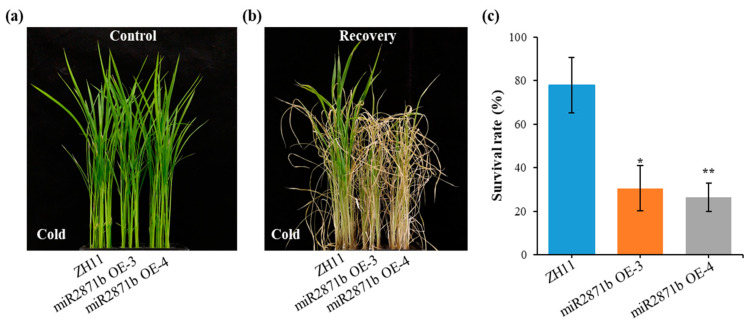
Reduced cold tolerance of miR2871b transgenic plants to cold stress. (**a**) Wild-type and transgenic rice plants before treatment. (**b**) Wild-type and transgenic plants recovery for 7 d. (**c**) The survival rates of wild-type and transgenic plants after cold stress. Data are means of three biological replicates, * *p* ≤ 0.05, ** *p* ≤ 0.01 using Student’s *t*-test.

To determine the potential involvement of miR2871b in cold stress response, the responses of transgenic and wild-type plants were cold-stressed. When the plants were cultured under control conditions, both transgenic and wild-type plants grew well, and there was no significant difference in the phenotype (Figure 3a). However, when the plants were kept at a low temperature (4 °C) for 5 d, the leaves of transgenic plants showed more severe curling and wilting symptoms than the wild-type plants. The plants were then subjected to recovery with culture solution for 7 d. The survival rates were significantly lower for transgenic lines miR2871b OE-3 (at 30.56%) and miR2871b OE-4 (at 26.39%) compared with those of the wild-type plants at 77.78% (Figure 3b,c). These results indicated that the transgenic plants were less tolerant to cold stress.

**Figure 4:**
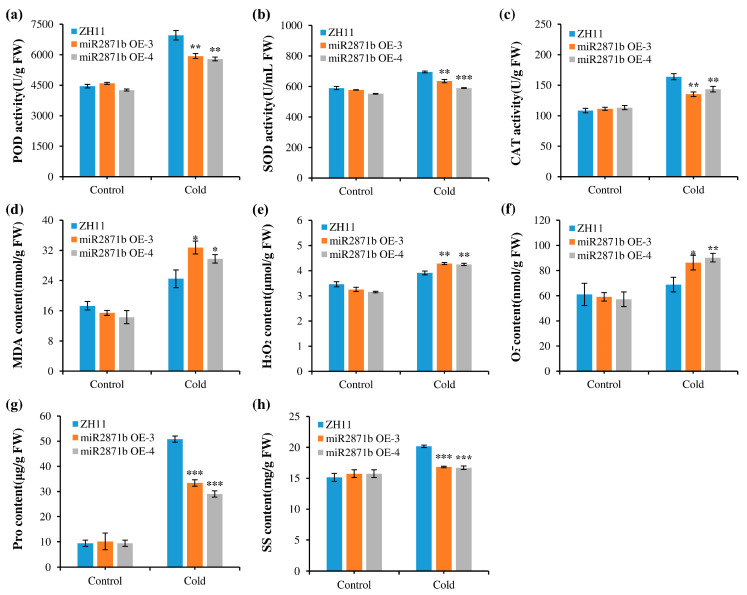
Effect of miR2871b overexpression on physiological indices in a cold environment. (**a**) POD activity, (**b**) SOD activity, (**c**) CAT activity, (**d**) MDA content, (**e**) H_2_O_2_ content, (**f**) O_2_^−^ content, (**g**) Pro content, (**h**) SS content. Data represented the means and standard errors of the triplicates. Data are means of three biological replicates, * *p* ≤ 0.05, ** *p* ≤ 0.01, *** *p* ≤ 0.001 using Student’s *t*-test.

To further elucidate the roles of miR2871b under cold stress conditions, the levels of physiological indicators and the activities of related enzymes were measured after 48 h of cold stress treatment. As shown in Figure 4, there were no significant differences in the contents of H_2_O_2_, O_2_^−^, MDA, Pro, and SS and the activities of POD, SOD, and CAT among all plants under control conditions. After cold stress treatment, the plants exhibited increased accumulation of O_2_^−^ and H_2_O_2_. Compared to the wild-type plants, transgenic plants had significantly higher O_2_^−^ and H_2_O_2_ content. The MDA content of the transgenic plants was significantly higher than that of the wild-type plants. This indicated that under cold stress, transgenic plants had higher levels of membrane injury than wild-type plants. In contrast, the activities of antioxidant enzymes (POD, SOD, and CAT) were significantly lower in transgenic plants than in wild-type plants. Free Pro- and SS-regulated osmotic pressure under stress and the contents of these two osmoregulatory substances in transgenic plants were significantly lower than those in the wild-type plants. These results indicated that compared with wild-type plants, miR2871b overexpressing plants had a weaker antioxidant capacity, higher degree of plasma membrane peroxidation, weaker ability to scavenge ROS, poorer osmoregulatory capacity, and were more sensitive to cold stress.

### 2.4. Overexpression of miR2871b in Rice Decreases Salt Tolerance

**Figure 5:**
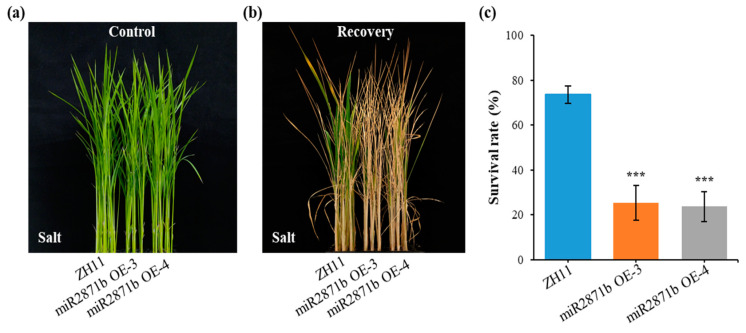
Reduced salt tolerance of miR2871b transgenic plants to salt stress. Plants grew to the three-leaf and one-cord stage and were treated with a nutrient solution supplemented with 150 mM NaCl for 7 d. (**a**) Wild-type and transgenic rice plants before treatment. (**b**) Wild-type and transgenic plants recovery for 7 d. (**c**) Survival rates of wild-type and transgenic plants after salt stress. Data are means of three biological replicates, *** *p* ≤ 0.001 using Student’s *t*-test.

Under normal conditions, there were no obvious abnormalities in the morphological development of the wild-type and miR2871b overexpressing plants (Figure 5a). To identify the salt stress tolerance of miR2871b overexpressing rice, we planted transgenic and wild-type plants in a nutrient solution supplemented with 150 mM NaCl. After one week, the leaves of transgenic plants exhibited severe wilting and stunting, whereas the leaves of wild-type plants remained largely healthy and continued to grow. After 7 d of salt-treated plants recovery, most wild-type plants remained healthy, whereas transgenic plants were severely wilted or stunted, and some plants died (Figure 5b). The survival rates were then counted, and the survival rate of wild-type plants was up to 73.61%, while those of miR2871b OE-3 and miR2871b OE-4 plants were only 25.35% and 23.61%, respectively (Figure 5c). These results indicated that miR2871b had a negative regulatory role on salt stress tolerance in rice.

**Figure 6:**
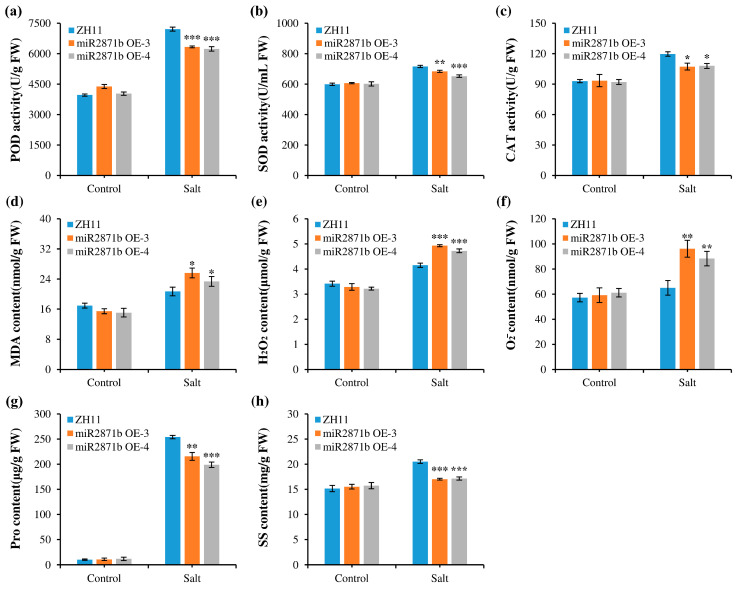
Effect of miR2871b overexpression on physiological indices of rice plants under salt stress. (**a**) POD activity, (**b**) SOD activity, (**c**) CAT activity, (**d**) MDA content, (**e**) H_2_O_2_ content, (**f**) O_2_^−^ content, (**g**) Pro content, (**h**) SS content. Data represented the means and standard errors of triplicates. Data are means of three biological replicates, * *p* ≤ 0.05, ** *p* ≤ 0.01, *** *p* ≤ 0.001 using Student’s *t*-test.

To analyze the reasons why transgenic plants performed worse under salt stress, physiological indices of the plants under normal and salt stress conditions for 48 h were determined. When grown under normal conditions, no differences were observed in the physiological indices among all plants. However, after salt stress treatment, the activities of POD, SOD, and CAT and the contents of Pro and SS in the transgenic plants were significantly lower than those in the wild-type plants, while the contents of H_2_O_2_, O_2_^−^, and MDA were significantly higher than those in the wild-type plants (Figure 6). The changes in these physiological indicators were consistent with those observed under cold stress. Therefore, it can be concluded that the overexpression of miR2871b had a significant attenuating effect on salt tolerance in transgenic rice.

### 2.5. Analysis of Differentially Expressed Genes in miR2871b Overexpressing Rice Plants

**Figure 7:**
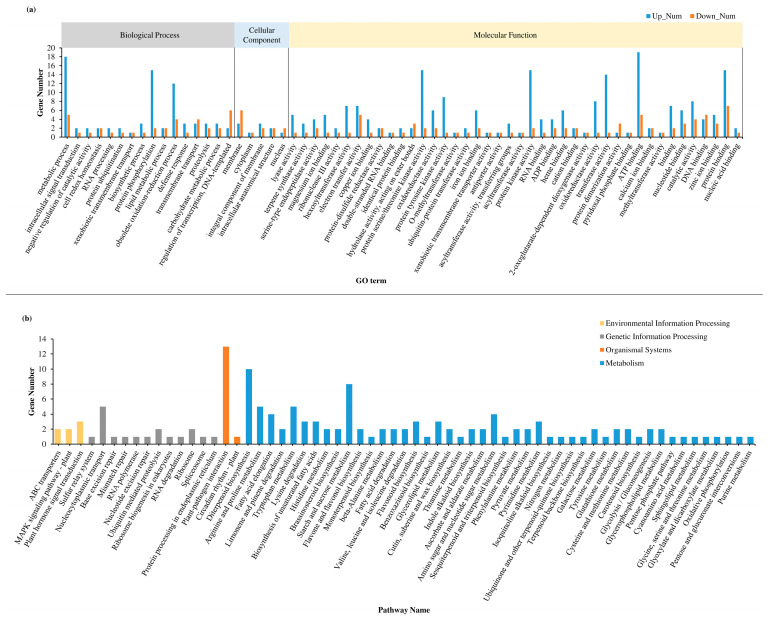
Analysis of differentially expressed genes in miR2871b transgenic plants. (**a**) GO classification of differentially expressed genes. (**b**) KEGG pathway analysis of differentially expressed genes.

Overexpression of miRNAs may lead to significant alterations in the expression of various related genes, consequently impacting the stress response of plants. To investigate the effect of overexpressing miR2871b on the gene network, we harvested the whole plants of wild-type and miR2871b OE-4 at the three-leaf and one-cord stage and performed transcriptome sequencing. Based on sequencing results, 266 significantly up-regulated genes, and 146 down-regulated genes were detected in the miR2871b overexpressing plants compared to the wild-type plants (Table S2). To understand the functions of these 412 DEGs, we conducted GO functional annotation of the DEGs to gain a better understanding of the effects of miR2871b overexpression on the stress response. In the GO analysis, the DEGs were annotated into three categories: biological process, cellular component, and molecular function (Figure 7a). Most of the DEGs were annotated with the functions related to metabolism, binding, and enzyme activity. In addition, some DEGs have been annotated with functions such as protein phosphorylation, biosynthetic process, response to stress, obsolete oxidation-reduction process, defense response, intracellular signal transduction, and others. Moreover, the most significantly enriched GO items were analyzed according to the *Q*-value, and “terpene synthase activity” and “lyase activity” were found to be the strongly enriched terms (Table S3). These results suggested that the overexpression of miR2871b impacted the expression of genes involved in various cellular processes associated with metabolism, biosynthesis, and stress responses.

In addition, the DEGs were annotated by KEGG pathways, which enhanced our understanding of the effects of miR2871b overexpression on rice plant pathways. The results showed that the functional hierarchy of annotated genes in the KEGG orthology system was divided into four categories: metabolism, genetic information processing, environmental information processing, and organismal systems (Figure 7b). Most annotated genes were related to metabolism, followed by genetic information processing. KEGG pathway analysis showed that the most significantly enriched pathway was “plant-pathogen interaction”. Additionally, DEGs were also annotated to several other pathways, including “diterpene biosynthesis”, “starch and sucrose metabolism”, “nucleocytoplasmic transport”, and “plant hormone signal transduction”. Moreover, the most significantly enriched KEGG pathways were analyzed according to the *Q*-value, and “diterpene biosynthesis” was found to be the most enriched pathway in the KEGG pathway annotation (Table S4). These data suggested that elevated miR2871b expression alters the expression of genes involved in various pathways, such as metabolism, signal transduction, and secondary metabolite biosynthesis, therefore affecting abiotic stress tolerance in rice plants. One caveat, however, is that only one overexpression line was examined for our transcriptome sequencing analysis. The insertion site or sites, because there might be multiple insertions of the overexpression construct in the transgenic line, can influence the result of transcriptome sequencing, especially if it is in an exon, which cannot be excluded.

### 2.6. Analysis of cis-Acting Elements and Target Prediction of miR2871b

A comprehensive understanding of the upstream *cis*-acting elements and downstream putative targets of miRNAs will greatly contribute to unraveling the mechanisms by which miRNAs regulate abiotic stresses. Various *cis*-acting elements in the promoter region may contain regulatory information on miRNA genes. To understand the *cis*-acting element components in the promoter of miR2871b, we extracted an approximately 1500 bp genomic sequence upstream of miR2871b of DXWR and blasted it against the PlantCARE database to search for *cis*-acting elements. Ten different types of potential *cis*-acting elements associated with environmental stress were identified (Table 1). Among these *cis*-acting elements, there were several elements involved in defense and stress responsiveness (TC-rich repeats), abscisic acid responsiveness (ABRE), and MeJA-responsiveness (CGTCA-motif, TGACG-motif). In addition, there were also light-responsive, gibberellin-responsive, and auxin-responsive elements. These results indicated that the induced expression of miR2871b may be mediated by various factors involved in plant stress response.

**Figure 8:**
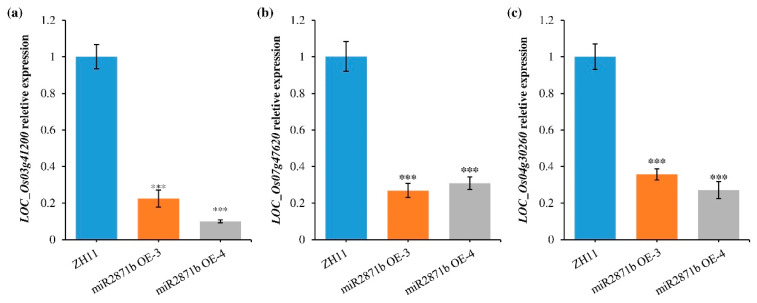
Expression analysis of miR2871b putative target genes in wild-type and transgenic plants. (**a**) *LOC_Os03g41200*. (**b**) *LOC_Os07g47620*. (**c**) *LOC_Os04g30260*. Data are means of three biological replicates, *** *p* ≤ 0.001 using Student’s *t*-test.

MiRNAs can participate in various biological processes by regulating the expression of target genes. Therefore, the identification and characterization of miRNA target genes are extremely significant for understanding their biological functions. To explore the regulatory targets of miR2871b, the psRobot website was used, and 709 putative candidates were identified (Table S5). These putative targets appeared to be involved in a wide range of biological processes, including those related to retrotransposon, transposon, transcription factors, calmodulin-binding, and disease resistance. MiRNAs usually negatively regulate the expression of target genes by degrading or inhibiting translation. Consequently, the target genes predicted by psRobot were compared with the down-regulated DEGs derived from transcriptome sequencing, and three candidate target genes were obtained, i.e., *LOC_Os03g41200*, *LOC_Os07g47620*, and *LOC_Os04g30260*. Of these, *LOC_Os03g41200* was predicted to encode a retrotransposon protein, *LOC_Os07g47620* was predicted to encode a universal stress protein domain-containing protein, and *LOC_Os04g30260* was predicted to encode OsWAK47- OsWAK receptor-like protein kinase. We further detected the expression levels of these three putative target genes in miR2871b overexpression plants by qRT-PCR. The results showed that the expression levels of all three genes were significantly down-regulated in the transgenic plants compared with the wild-type plants (Figure 8), which was consistent with the transcriptome sequencing results.

## 3. Discussion

MiRNAs participate in plant growth, development, and response to biotic and abiotic stresses [38,39]. The characterization of miRNAs involved in the adaptation to stress conditions and the elucidation of their molecular mechanisms will contribute to a deeper understanding of plant stress response mechanisms and signaling pathways. Previous studies have found miR2871b was significantly down-regulated under cold stress conditions in DXWR [37], and miR2871b was also found to be significantly down-regulated after salt stress in the salt-tolerant rice accession Pokkali [40]. Furthermore, Barera-Figueroa et al. [14] using high-throughput sequencing found that miR2871b can be induced by drought, cold, and salt stress in rice inflorescence. These studies suggested that miR2871b likely is involved in the rice stress response. In this study, the expression pattern of miR2871b was analyzed in DXWR which is a common wild rice with excellent agronomic traits and tolerance to biotic and abiotic stresses [41], and found that the expression of miR2871b was significantly down-regulated under cold and salt stress. These findings implied that miR2871b may be associated with the stress response in DXWR. However, to date, the role of miR2871b in stress response remains elusive. This prompted us to conduct further experiments to characterize the function of miR2871b in stress response, as well as to understand the molecular mechanism by which miR2871b regulates cold and salt tolerance. Overexpression of miRNAs is useful in analyzing the function of stress-responsive miRNAs in the plant stress response [42,43]. In the present study, miR2871b was overexpressed and the transgenic plants were observed to have significantly reduced cold and salt tolerance, suggesting that miR2871b is a negative regulator of cold and salt stress tolerance.

The ability of plants to tolerate adverse stress conditions can be inferred from the changes in various physiological indicators. Presently, a multitude of studies have been undertaken to understand stress tolerance by detecting physiological indicators, including ROS, MDA, and antioxidant enzymes [44,45]. The examination of diverse physiological indicators after stress is crucial for comprehending the regulatory roles of miR2871b in the stress response. When plants are exposed to severe stress conditions, there is a substantial accumulation of ROS. High concentrations of ROS cause lipid peroxidation, MDA accumulation, and cell membrane damage [46,47]. MDA, a byproduct of lipid oxidation in plant cell membranes, serves as a significant indicator of the degree of oxidative damage caused by ROS [48]. Our study found that the levels of MDA, H_2_O_2_, and O_2_^−^ in the miR2871b overexpressing plants were significantly higher than those in the wild-type plants under salt and cold stress conditions, suggesting that oxidative damage was more serious and decreased stress tolerance in transgenic plants. Despite the detrimental effects of elevated levels of ROS on plants, they possess the ability to counteract oxidative damage through the synthesis of diverse antioxidant enzymes and antioxidants [49,50]. This study found that SOD, POD, and CAT activities of both transgenic and wild-type plants increased after cold and salt stress; however, the enzyme activities in transgenic plants were significantly lower than those in wild-type plants. In addition, the accumulation of osmoprotectants, such as free Pro and SS, can directly protect plants from various stresses [51,52]. In this study, the Pro and SS contents in the miR2871b transgenic plants were lower than in the wild-type plants under cold and salt stress conditions. These results suggest that the overexpression of miR2871b may reduce the ability of rice plants to maintain ROS at a suitable level, resulting in severe oxidative damage in transgenic plants.

Meanwhile, transcriptome sequencing has been widely used to study the growth and development patterns, stress physiology, stress response mechanisms, and metabolic regulatory networks of plants [53,54,55,56]. In this study, transcriptome sequencing was conducted on the wild-type plants and miR2871b overexpression plants to obtain a more comprehensive understanding of the regulation of miR2871b. A total of 412 DEGs were identified, which have the potential to significantly impact various metabolic activities and cellular processes in response to adverse conditions in rice. These results suggest that miR2871b overexpression in rice plants extensively alters gene expression and affects cold and salt tolerance by regulating stress-related metabolic pathways.

Furthermore, the elucidation of the roles of miRNAs in plant stress responses will be facilitated by the identification of specific upstream regulatory elements. The *cis*-acting elements present on the miRNA promoter possess the ability to identify and interact with distinct transcription factors, therefore leading to the regulation of miRNA transcription in a spatiotemporal-specific manner [57]. Notably, upstream *cis*-acting regulatory element analysis of miR2871b revealed the presence of stress response-related elements such as ABRE and TC-rich repeats. ABRE is a conserved *cis*-acting element in the promoter region, through which abscisic acid (ABA) activates the expression of downstream genes [58]. ABA is a plant hormone that is essential for plant growth and integrates various stress signals to control subsequent stress responses [59]. This suggests that miR2871b regulation of abiotic stress may be influenced by the ABA signaling pathway. Furthermore, other phytohormones, including methyl jasmonate (MeJA) and gibberellins (GA), also play an important role in cold and salt responses [60,61,62]. In addition, the TC-rich repeat element is a well-recognized *cis*-acting element associated with plant defense and adversity [63]. According to previous studies, TC-rich repeat elements have been found in the promoters of various stress-related miRNAs, such as those related to cold, salt, and drought stress [27,64,65]. The existence of stress-responsive *cis*-elements provides evidence of the importance of miR2871b in the regulation of cold and salt stress response networks.

MiRNAs generally execute biological functions via the negative regulation of specific target genes. In plants, miRNAs exhibit perfect or near-perfect complementary pairing with their target gene mRNAs [66]. This feature of plant miRNA cleaving target genes predicts plant miRNA targets by programmed homology query algorithms possible [67,68]. In this study, we predicted 709 possible targets for miR2871b using psRobot software (version 1.2) and found that the putative targets were involved in a wide range of biological processes. Additionally, we screened three candidate target genes *LOC_Os03g41200*, *LOC_Os07g47620*, and *LOC_Os04g30260* using transcriptome sequencing data and psRobot data. The functional annotation of *LOC_Os03g41200* was related to retrotransposons. Retrotransposons are widely present in the genomes of higher plants and are closely associated with plant stress resistance [69]. Several retrotransposons have been found to affect the response of plants to cold, heat, and salt stress [70,71,72]. *LOC_Os07g47620* was annotated as a universal stress protein domain-containing protein (USP). It has been found that USP can act in several cellular signaling transducers and metabolic regulators and has a wide range of biological functions to withstand a variety of biotic and abiotic stresses [73,74,75]. *LOC_Os04g30260* was predicted to encode OsWAK47- OsWAK receptor-like protein kinase. *OsWAKs* are a class of receptor-like kinase genes that span cell membranes and enable cells to recognize and respond to a multitude of extracellular environments [76]. Further efforts to unravel the functions of these putative targets and *cis*-acting elements of miR2817b will be particularly important to gain new insights into its regulatory mechanisms.

## 4. Materials and Methods

### 4.1. Plant Material and Stress Treatments

The seeds of DXWR originated from Dongxiang County, Jiangxi Province, China, and were stored in our laboratory at Jiangxi Normal University, China. Cultivated rice *japonica* accession Zhonghua 11 (ZH11) was used for transgenic analysis. The seeds were soaked in distilled water at 32 °C for 48 h to induce germination. Germinated seeds were transplanted into 96-pore plastic hydroponic boxes (87 mm wide, 127 mm long, 114 mm high, and 5 mm pore size) containing IRRI (International Rice Research Institute, Los Banos, Philippines) nutrient solution. Then, the plants were planted in a greenhouse with IRRI nutrient solution in the boxes under the following growth conditions: 14 h light at 28 °C/10 h dark at 25 °C, with a relative humidity greater than 80%.

To analyze the expression profile of miR2871b under abiotic stress, DXWR seedlings at three-leaf and one-cord stage were exposed to the following treatments: cold (4 °C) and salt (200 mM NaCl) stresses. Shoot and root were sampled at 0, 1, 4, 8, 12, and 24 h after treatment, each consisting of three individual plants, then immediately frozen in liquid nitrogen and stored at −80 °C. To investigate the impact of miR2871b overexpression on cold and salt tolerance, we conducted stress treatments using wild-type plants and two transgenic lines. Twenty-four seedlings of each sample were planted in the 96-pore plastic hydroponic boxes for stress treatment, and three biological replications were performed for different stress treatments. For the cold stress treatment, rice seedlings were placed under cold conditions (14 h light/10 h dark at 4 °C) for 5 d. For the salt stress treatment, rice seedlings were transferred to a nutrient solution containing 150 mM NaCl and allowed to grow for 7 d. After stress treatment, rice seedlings were transferred to normal conditions for one week to recover, and the number of surviving seedlings was counted.

### 4.2. Plasmid Construction and Genetic Transformation

To generate transgenic rice plants overexpressing miR2871b of DXWR, a 910 bp fragment including the miR2871b stem-loop structure was amplified from DXWR genomic DNA via PCR with specific primers miR2871b-F/R (Table S1). The amplicon was sequenced and cloned into the *Kpn*I and *Sal*I restriction enzyme recognition sites of the binary vector 35S-pCAMBIA1300, resulting in an overexpression vector. Then, the recombinant overexpression vector was transferred to the *Agrobacterium tumefaciens* strain EHA105, which was subsequently transformed into ZH11 [77]. Transgenic rice plants carrying DXWR miR2871b overexpression vector were confirmed via PCR using the specific primers Det-miR2871b-F/R (Table S1). Det-miR2871b-F was designed on the promoter region of the overexpression vector, while Det-miR2871b-R was designed on the miR2871b overexpression fragment.

### 4.3. Physiological Index Determination

Unstressed (control) and stressed 48 h samples of wild-type and transgenic lines were collected for physiological analysis. Samples were finely ground into powder with a pestle in liquid nitrogen and stored at −80 °C for measurement of different physiological indicators. The experimental details of the determination of peroxidase (POD), superoxide dismutase (SOD), and catalase (CAT) activities have been described previously [78,79]. The malondialdehyde (MDA) content was determined using the thiobarbituric acid (TBA) method [80]. The concentrations of reactive oxygen species (ROS), including hydrogen peroxide (H_2_O_2_) and superoxide anions (O_2_^−^), were determined following the previous method [81]. Free proline (Pro) content was estimated using ninhydrin colorimetry [82]. Soluble sugar (SS) content was determined using anthrone colorimetry [83].

### 4.4. RNA Isolation and qRT-PCR Analysis

Total RNA was isolated using TRIzol reagent (Invitrogen, Carlsbad, CA, USA) according to the manufacturer’s instructions. The purity and concentration of RNA were measured using a NanoDrop 2000 spectrophotometer (Thermo Fisher Scientific, Lenexa, KS, USA). To analyze the expression of miR2871b, reverse transcription was performed using the miRNA 1st Strand cDNA Synthesis Kit (by stem-loop) (Vazyme, Nanjing, China). To analyze the target gene expression, mRNAs were reverse transcribed using the Primescript™ RT reagent Kit with gDNA Eraser (Takara, Dalian, China). qRT-PCR was performed on an ABI7500 Real-time system (Applied Biosystems, Foster City, CA, USA) using the TB Green Premix Ex Taq II (Tli RNaseH Plus) Kit (Takara, Dalian, China). *U6* and *OsActin1* were used as internal reference genes to correct for the relative expression levels of miRNAs and mRNAs, respectively [84]. All reactions were performed in triplicate for each sample. The relative expression levels were calculated using the 2^−ΔΔCt^ method [85,86]. All primers used in this study are listed in Table S1.

### 4.5. Transcriptome Sequencing and Bioinformatic Analysis

For transcriptome sequencing, the whole transgenic and wild-type plants at the three-leaf and one-cord stages were collected. Three biological replicates were collected for each sample and immediately frozen in liquid nitrogen. Thereafter, cDNA library construction and transcriptome sequencing of the six samples were performed by LC-Bio Technology Co., Ltd. (Hangzhou, China). Raw reads were processed using Cutadapt (version: cutadapt-1.9) [87] to trim adapter sequences and filter out low-quality reads to obtain high-quality clean reads. The HISAT2 (version: hisat2-2.2.1) package [88] was used to map the reads to the rice reference genome (http://rapdb.dna.affrc.go.jp/download/irgsp1, accessed on 10 November 2022). Transcripts were reconstructed using Stringtie software (version: stringtie-2.1.6), and expression levels were calculated for all genes in each sample [89]. The mRNA expression levels were quantified using FPKM (Fragments Per Kilobase Million) [90]. Differential expression analysis was performed using DESeq2 software (version: release 3.17) [91] between the wild-type and transgenic samples. Genes with a false discovery rate (FDR) < 0.05 and absolute fold change ≥ 2 were considered differentially expressed genes (DEGs) [92]. Thereafter, the DEGs were subjected to enrichment analysis of GO functions and KEGG pathways.

### 4.6. Putative Target Prediction and Analysis of Cis-Acting Elements

Target prediction of miR2871b was performed using a plant small RNA analysis toolbox, psRobot, with default parameters and score ≤ 4 [65,93]. The mature sequence of miR2871b is TATTTTAGTTTCTATGGTCAC. The next-generation sequencing data of DXWR can be available in the NCBI SRA database with ID SRP070627. The mature sequence of miR2871b was used as the input sequence and MSU Rice Genome Annotation (version 7) was used as the library for the target search. For *cis*-acting element analysis, a ~1500 bp DNA sequence upstream of miR2871b was isolated from the DXWR genome. PlantCARE software (https://bioinformatics.psb.ugent.be/webtools/plantcare/html/ accessed on 23 December 2022) was used to identify putative *cis*-acting elements in the miR2871b promoter [94].

## 5. Conclusions

The phenotype and various physiological indices measurement showed that miR2871b, as a negative regulator, affected cold and salt tolerance in rice. Furthermore, the *cis*-acting elements and target genes of miR2871b were predicted and analyzed, and 10 critical elements and three candidate target genes were identified. Transcriptome sequencing-based identification of 412 DEGs in plants overexpressing miR2871b, which exhibit a wide range of biological functions and are involved in multiple pathways. The results derived from these experiments provide novel information for further research and understanding of the molecular mechanisms of miRNA-associated cold and salt stress tolerance.

## Figures and Tables

**Table 1:** Stress-related *cis*-element analysis of miR2871b.

Site Name	Loc (Strand)	Sequence	Function
ABRE	−1314 (+); −1437 (−)	ACGTG	involved in the abscisic acid responsiveness
Box 4	−11 (+)	ATTAAT	part of a conserved DNA module involved in light responsiveness
CGTCA-motif	−702 (−); −705 (+)	CGTCA	*cis*-acting regulatory element involved in the MeJA-responsiveness
GARE-motif	−215 (+)	TCTGTTG	gibberellin-responsive element
G-box	−1313 (+); −1437 (+)	TACGTG; CACGTT	*cis*-acting regulatory element involved in light responsiveness
P-box	−1371 (−)	CCTTTTG	gibberellin-responsive element
TATC-box	−1280 (−)	TATCCCA	*cis*-acting element involved in gibberellin-responsiveness
TC-rich repeats	−369 (+)	ATTCTCTAAC	*cis*-acting element involved in defense and stress responsiveness
TGACG-motif	−702 (+); −705 (−)	TGACG	*cis*-acting regulatory element involved in the MeJA-responsiveness
TGA-element	−109 (+)	AACGAC	auxin-responsive element

## Data Availability

The data presented in this study are available in the article or Supplementary Material.

## Supplementary Materials

The following supporting information can be downloaded at: https://www.mdpi.com/article/10.3390/ijms241914502/s1.
