# Supplementary material for: MicroRNA2871b of Dongxiang Wild Rice (Oryza rufipogon Griff.) Negatively Regulates Cold and Salt Stress Tolerance in Transgenic Rice Plants
Source: Int J Mol Sci. 2023 Sep 25;24(19):14502. doi: 10.3390/ijms241914502 (PMC10572564; doi:10.3390/ijms241914502)
Supplement: Supplementary file 1 [file ijms-24-14502-s001.zip › Table S1.pdf]

Table S1. The primers used in this study

Primers	Sequence (5'-3')
miR2871b-F	ACAGGTACCGTGATCGATATTAAGCCGTATG
miR2871b-R	ACAGTCGACACGTGCTTTCAATGCTTGTG
HYG-F	CGAGAGCCTGACCTATTGCAT
HYG-R	CTGCTCCATACAAGCCAACCAC
Det-miR2871b-F	TCCCACTATCCTTCGCAAGAC
Det-miR2871b-R	GCCATCACGATGAGGCATGA
miR2871b-RT primer	GTCGTATCCAGTGCAGGGTCCGAGGTATTCGCACTGGATAC GACGTGACC
miR2871b-qF	GCCGCCCTATTTTAGTTTCTAT
miR2871b-qR	GTGCAGGGTCCGAGGT
LOC_Os03g41200-qF	TGTGGGCCGATCGTCAAAAT
*LOC_Os03g4120*0-qR	TGACAATACATCCACACAAAGGA
LOC_Os07g47620- qF	ACTGCTCCGTCATGATCGTC
LOC_Os07g47620- qR	AGAAGCTGCTGCTACCCAAG
LOC_Os04g30260- qF	TGATCATTACCGTGGGCGAG
LOC_Os04g30260- qR	GTCCATTGAGCGAGTCCACA
U6-F	CGATAAAATTGGAACGATACAGA
U6-R	ATTTGGACCATTTCTCGATTTGT
OsActin1-F	GTATCCATGAGACTACATACAACT
OsActin1-R	TACTCAGCCTTGGCAATCCACA
